# Challenges and Solutions for Designing and Managing pHealth Ecosystems

**DOI:** 10.3389/fmed.2019.00083

**Published:** 2019-04-18

**Authors:** Bernd Blobel

**Affiliations:** ^1^Medical Faculty, University of Regensburg, Regensburg, Germany; ^2^eHealth Competence Center Bavaria, Deggendorf Institute of Technology, Deggendorf, Germany; ^3^First Medical Faculty, Charles University Prague, Prague, Czechia

**Keywords:** pHealth, ecosystem, architecture, modeling, interoperability, knowledge representation, knowledge management

## Abstract

For improving quality and safety of healthcare as well as efficiency and efficacy of care processes, health systems turn toward personalized, preventive, predictive, participative precision medicine. The related pHealth ecosystem combines different domains represented by a huge variety of different human and non-human actors belonging to different policy domains, coming from different disciplines. Those actors deploy different methodologies, terminologies, and ontologies, offering different levels of knowledge, skills, and experiences, acting in different scenarios and accommodating different business cases to meet the intended business objectives. Core challenge is the formal representation and management of multiple domains' knowledge. For correctly modeling such systems and their behavior, a system-oriented, architecture-centric, ontology-based, policy-driven approach is inevitable, thereby following established Good Modeling Best Practices. The ISO Interoperability Reference Architecture model and framework offers such approach. The paper describes and classifies the ongoing paradigm changes. It presents requirements and solutions for designing and implementing advanced pHealth ecosystems, thereby correctly adopting and integrating existing pHealth interoperability standards, specifications and projects.

## Introduction

Starting in the nineties of the last century and speeding up during the last 10 years, healthcare systems undergo organizational and methodological paradigm challenges. That way, they respond to the challenges for increasing safety and quality of care as well as efficiency and efficacy of care processes under the known demographic, social, and economical constraints. The organizational paradigm change concerns the transition from organization-centric through disease-specific process-controlled (Disease Management Programs—DMPs) to person-centric care. The methodological paradigm change deals with the transition from a generalized through a specialized toward an instantiated approach. It starts with the phenomenological perspective of general care addressing health problems with one solution fitting all patients, followed by dedicated care for patient groups resulting from the stratification of population for specific clinically relevant conditions. This transition now evolves to personalized, preventive, predictive, participative precision medicine (P5M) considering individual health status, conditions, genetic, and genomic dispositions as well as social, occupational, environmental, and behavioral context, thus turning medicine from reactive to proactive (1–4). The described different levels of granularity and complexity of the considered health system are connected to different maturity levels of the underlying processes, i.e., the transition from empiric through evidence-based to translational medicine.

Alternatively, we can classify that paradigm change as transition from an observational through an analytical to a systems medicine approach, or in other words the transition from art to science. Such approach requires cooperation of many different and sovereign stakeholders from multiple domains. Involved disciplines include medicine and public health, natural sciences, engineering, administration, but also social and legal sciences and the entire systems sciences world (systems medicine, systems biology, systems pathology, etc.). System actors are any type of principals (person, organization, device, application, component, object) according to the Object Management Group's (OMG's) definition (5). The advancement of the methodological paradigm requires an advanced organizational paradigm.

Systems theory provides another formal way to describe health systems transformation. It offers an approach to reality by abstracting from all specialties and just focusing on those aspects of a real system the investigator is interested in, thereby providing a model of reality representing the real system's behavior, i.e., functionality. A system is an ordered composition of interrelated elements, separated from, and interacting with, its environment. A system's architecture covers its components, their functions and relations. The rules controlling the behavior of a system are summarized as policies of that system. The black-box system model describes the system functions based on the analysis of the system's output in relation to inputs and modifying conditions, so representing the empiric or phenomenological approach to medicine. The quality of input and output could belong to one of the three categories: material, energy, or information (6). The white-box approach of system theory investigates structure, functions and interrelations of the system's components for understanding and predicting the system's behavior, that way corresponding to the systems medicine approach of personalized health. Philosophers discussed this approach as the transition from describing the world toward understanding and changing the world.

To enable the described advanced knowledge-based interoperability between involved domains and actors, an agreed framework for representation, harmonization and implementation of related concepts, skills and abilities is inevitably required. Such a framework is the focus of the paper, which summarizes a series of related contributions by the author.

**Figure 1:**
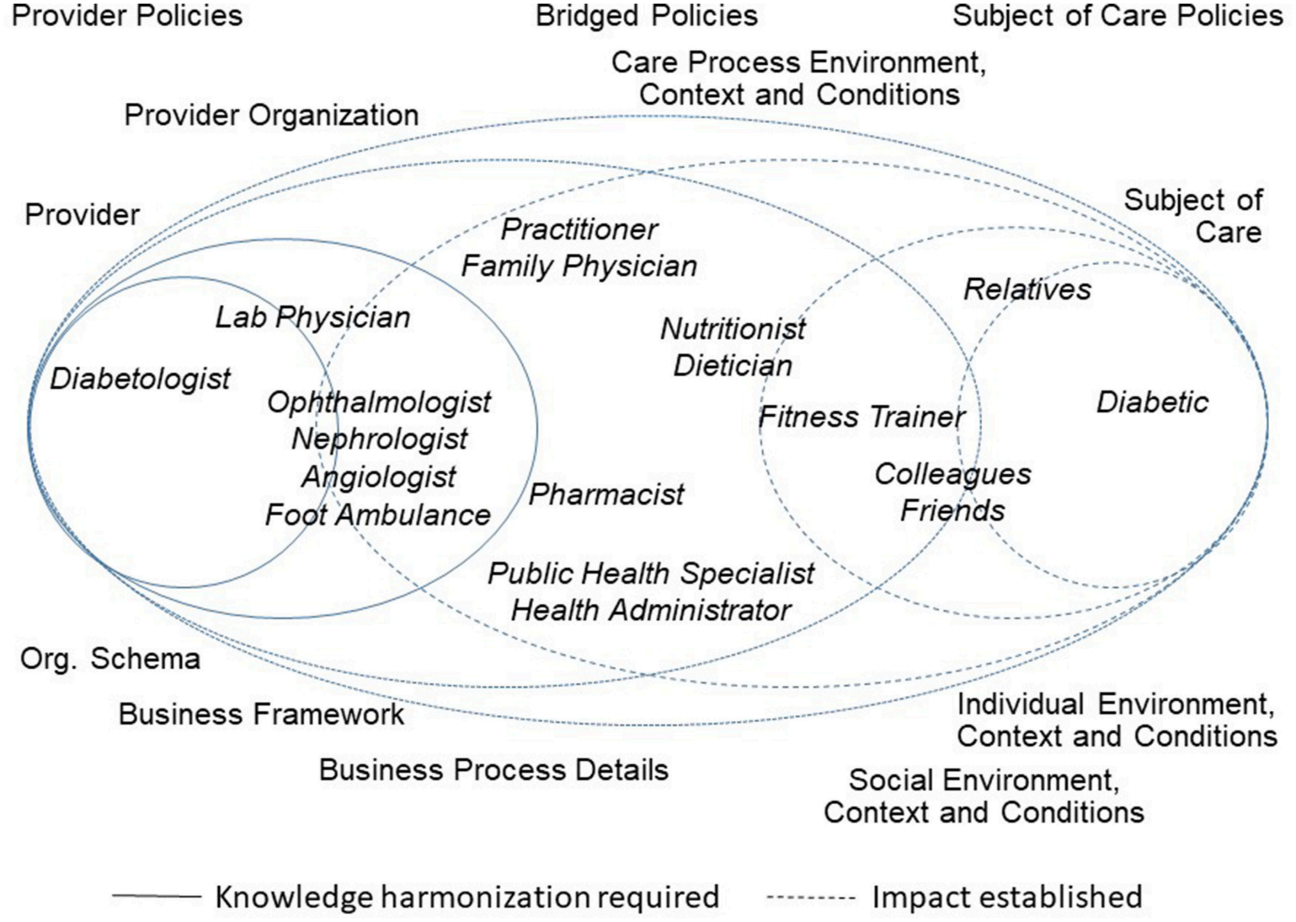
Paradigm changes in healthcare ecosystems.

Figure 1 presents the healthcare ecosystem, demonstrating the paradigm change with a focus on the system architecture including underlying policies. It exemplifies domain experts from (knowledge) domains involved in Type 2 Diabetes to facilitate the figure's understandability.

## Personal Health in the Light of P5M

The need for individualized care is not a new insight. Despite the statistical validation of the outcome provided by purpose-specific, clinical studies for stratified population, pathogenesis and/or efficiency of different therapies vary among patients with the same diagnosis. Therefore, good medical practice is based on three pillars: (a) the knowledge gathered by domain experts during the evolution of medicine and related sciences as well as from emerging projects and insights, (b) the practicing clinician's experience in interpreting and applying this knowledge, and (c) the consideration of the patient's individual context and conditions, weighted in that order. Empowered subjects of care gather knowledge through new technologies and social business and actively as well as passively provide data and information, thereby increasingly strengthening the third pillar. The personalized health approach just attaches a greater weight to personal pathogenesis and corresponding individual diagnoses and therapies, i.e., specializes and individualizes medicine toward the patient, His/her context, conditions, and preferences, thereby understanding the individual molecular and cell-specific reasons for, and predicting or better even preventing, the development and course of a disease (Personalized Medicine Coalition) (7). The ability of single cell analysis, the measurement of sub-cell components allows the assessment of cell mechanisms being normal or disease-related. A concrete example is the molecular genomics analysis of diseases by combining individual genomes with phenotypic information. P5M considers beside the genome also epigenome, proteome, transcriptome, metabolome, microbiome, pharmacome, cognitive-affective behaviorome, nutrition, and other factors for creating a complex description of interactions among all components and factors, also called interactome. The outcome may serve as basis for predictive models of human health, reflecting the dynamics of diseases and so enabling prediction and decision support (1). Another component deployed for P5M are biomarkers as “a sign of a normal or abnormal process, or of a condition or disease” (8). For more details, see Blobel (9). Requiring the inclusion of a huge amount of information about the patient in his/her impacting contextual framework, personalized health considers the entire spectrum from elementary particles through atoms, single elements, anorganic compounds, organic basic elements, macromolecules, macromolecule complexes, cell organelles, cells, tissues, organs, body, and population up to society (6).

The transformation of medicine described above is strongly impacted and supported by technological paradigm changes. Here, not just imaging advancing toward 3D, 4D, and 5D methodologies and the integration of machine learning, but also distributed systems evolving toward container models and micro-services, mobile technologies, nano- and molecular technologies, knowledge representation (KR) and knowledge management (KM), artificial intelligence (AI), robotics, bioinformatics, big data and prescriptive analytics, natural language processing (NLP), cloud computing, social business, cognitive computing, and finally the Internet of Things (IoT) must be mentioned. By improving the systems' intelligence, good practices, advanced knowledge and skills can be shared, professionals and lay people (including the subject of care) with different level of qualification and skills can be integrated in the care delivery chain, making best solutions broadly available and financially sustainable (10).

Beside AI and predictive analytics, nano-biotechnologies such as nano-biosensors (biochips, lab-on-a-chip) or nano-fluids are essential tools for diagnosis in personalized medicine. Regarding nano-biotechnologies for therapy, molecular self-assembly mechanisms, intelligent drug delivery systems, and nano-machines enter the market. Biosensors are devices used to detect fact and level of presence of a biological analyte externally or internally to the human body (11). Wearable (or even implantable) sensor devices detect and assess psychophysiological processes (12). An overview on methodologies and technologies needed for pHealth is presented in Blobel (9).

## Challenges in Describing the pHealth Ecosystem

There are different terms established around pHealth and partially synonymously used such as person-centric health, individualized health, personal health, personalized health, precision medicine, mobile health, pervasive health, ubiquitous health, etc. More details can be found in Blobel et al. (13). First, some definitions should be introduced to harmonize views and understanding amongst the readers of this paper.

Following the specifications given in the former sections, pHealth, or personalized health allows to respond to the subject of care's unique needs. Hence, pHealth is usually defined as individually tailored health services for prevention and lifestyle, diagnosis, and treatment based on the individual assessment of personal health risks according to the individual health status, individual as well as family history, environmental and social context of the subject of care, thereby using its genetic, proteomic, anatomical, physiological, and any other clinical as well as biological information. A similar but shorter description is provided by the American Medical Association (AMA) defining pHealth as “healthcare that is informed by each person's unique clinical, genetic, genomic, and environmental information” (14). pHealth deploys specific biomarkers, genetics, genomics, regenerative medicine and stem cell technology, liquid biopsies, etc. It exploits among others wearable and implantable sensors and actuators, bio- and molecular technologies, data integration and analytics, artificial intelligence as well as social businesses.

The pHealth system covers the organization of people, institutions, and resources that deliver pHealth services meeting the health needs of individuals. The pHealth ecosystem describes the aforementioned system and its environment it interrelates with. More explicit, the World Health Organization (WHO) described a health-related ecosystem as combined physical and biological components of an environment, forming complex sets of relationships and function as a unit as they interact with their physical environment (15).

According to Alter, a model is a partial representation of reality restricted to attributes the modeler is interested in according to the purpose of modeling, the addressed audience, etc. (16). A purpose of models is to create and/or to represent knowledge. In that sense, Langhorst et al. (17) defined a model as an unambiguous, abstract conception of some parts or aspects of the real-world corresponding to the modeling goals. They requested that the relevant stakeholders shall define the provided view of the model. This includes the way of structuring and naming the concepts of the problem space. First capturing key concepts and key relations at a high level of abstraction, different abstraction levels should be used iteratively. However, the first iteration must be performed in a top-down manner to guarantee the conceptual integrity of the model (17). The described approach meets good modeling design principles such as orthogonality, generality, parsimony, and propriety (18). Many health interoperability specifications, projects, and even standards model the system in questions just at the data model or information model level [dimension 1 and 2 of the modeling dimensions after Krogsti (19)], slowly evolving toward the medical domain's knowledge representation [dimension 3 according to Krogstie (19)] by referencing SNOMED-CT concepts and terms. P5M however requires the management and integration of the interdisciplinary knowledge space of the domains involved, representing dimension 4 after (19).

## Modeling pHealth Ecosystems

**Figure 2:**
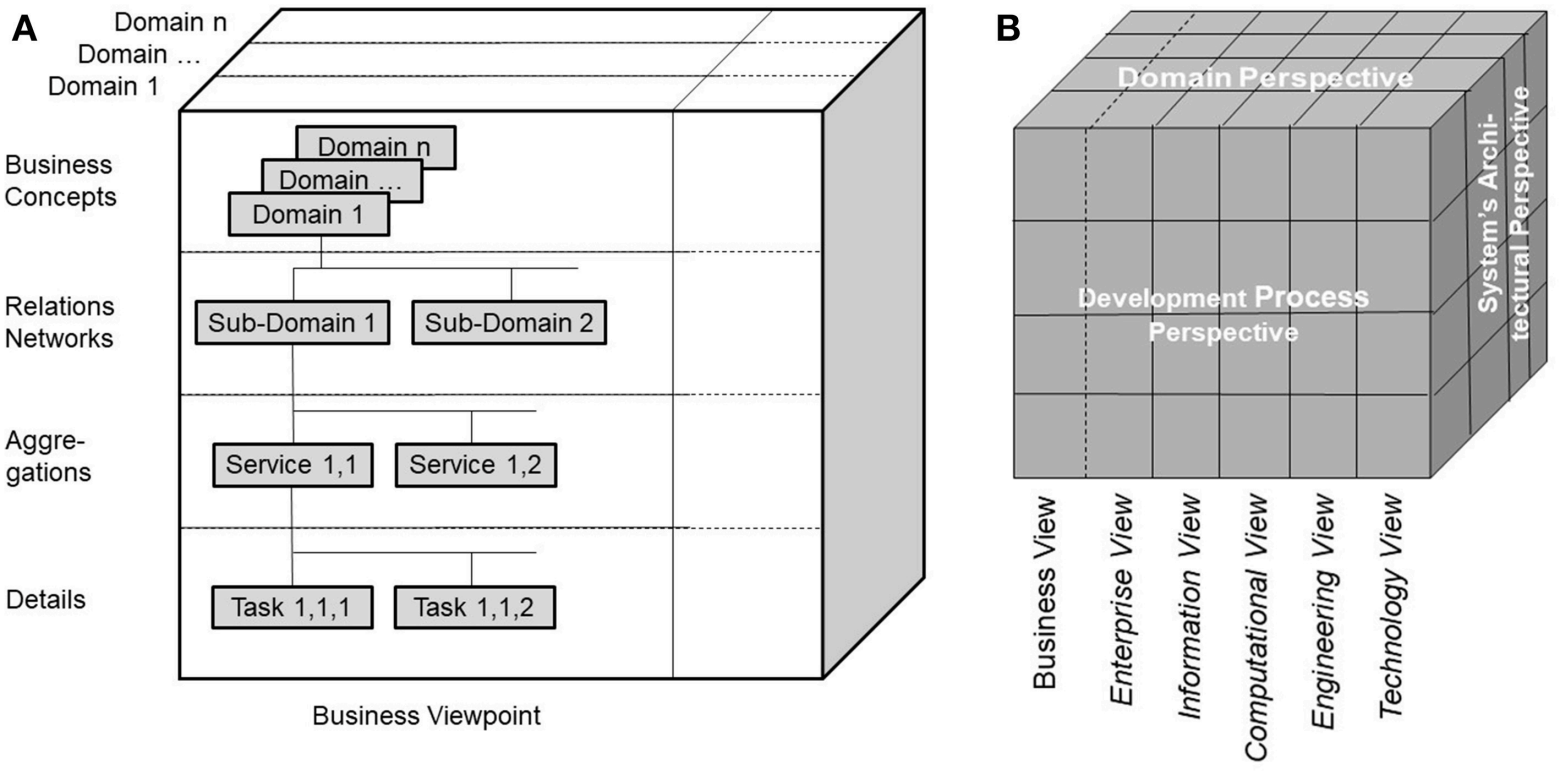
**(A)** Architectural representation of pHealth ecosystems, **(B)** ISO Interoperability Reference Architecture.

Modeling pHealth systems as described in the former sections puts special burden on designers and implementers to enable communication and cooperation among experts from different domains involved in pHealth using their specific methods, terminologies and underlying ontologies. The representation of the system covering the subject of care and the processes of analyzing and managing his or her health must consider all levels of its structural granularity and related behavioral aspects. The structural elements comprise elementary particles, atoms, molecules, cell components, cells, tissues, organs, bodies, communities and finally the population. Dealing with functions of, and relationships between, the system's elements from a disciplinary or domain perspective, we have to investigate quantum-mechanical effects in the atomic and subatomic world, biochemical processes, interrelations based on classical physics, and finally social interrelations in the macro-world. For consistently modeling pHealth ecosystems showing the aforementioned structural and functional spectrum, we have to restrict the system' complexity by focusing just on parts of the system when considering higher granularity levels. The solution to that problem is the abstraction from those domains' specificities, just describing the system's architecture. For that purpose, the Generic Component Model (GCM) has been developed in the mid-nineties (20, 21), which can be recursively used to manage the system's complexity. The outcome is a business system model, generically describing the architecture of systems according to the modelers' interests at a manageable level of complexity, thereby creating the composition/decomposition or generalization/specialization dimension of the system model. The top granularity level is the business system to be modeled. The first decomposition/specialization level “Relations Network” defines possible subsystems represented by sub-ontologies as explained below. At the next decomposition/specialization level “Aggregations,” components realizing specific services are specified, which can be refined at the next level of “Details” (Figure 2A). As the system's components relevant from special domains' perspectives may differ, the system's component model has to be refined for the domain-specific subsystems, creating the domain dimension of the system model (Figure 2A).

The next challenge is the representation and management of the knowledge defining structure and behavior of the system and its different perspectives or aspects by formally representing architecture and policies of the system and its subsystems.

Alter defines knowledge as “a combination of instincts, ideas, rules, and procedures that guide actions and decisions” (16). Knowledge of a domain of discourse (discipline), representing that domain's perspective on reality to facilitate reasoning, inferring, or drawing conclusions, is created, represented, and maintained by domain experts using their methodologies, terminologies and ontologies. Initiated by cognitive sciences, KR and KM happen on three levels: the epistemological (cognitive and philosophical) level, the notation (formalization and conceptual) level, and the computational or implementation level (22). Deploying KR techniques such as frames, rules, tagging, and semantic networks, a good KR has to manage both declarative and procedural knowledge. Gruber defined an ontology as formal explicit specification of a shared conceptualization of a domain of interest, that way describing an ordering system of entities of a domain and their relations (23). A concept is a knowledge component that shall be uniquely identifiable, independently accepted by experts and users. It has a representation and can be specialized and generalized as any component can (24). As discussed in Blobel (25–27), knowledge can be represented at different level of abstraction and expressivity, ranging from implicit knowledge up to fully explicit knowledge representation, i.e., from natural language up to universal logic. Expressivity is key for selecting an appropriate KR. While a more expressive knowledge representation language enables an easier and more compact expression of knowledge within the KR semantics and grammar, it is likely to require more complex logic and algorithms to construct equivalent inferences. This property results in the complexity problem of formal language and reasoning systems with the lack of computability, at the same time losing the consistency of the language system. In other words, highly expressive KRs are less likely to be complete and consistent, while less expressive KRs may be both complete and consistent. In summary, natural languages are not only efficient in representing meaning, shared knowledge, skills, and experiences assumed. They also provide an optimum between restriction to special structure and generative power enabling the rich and nevertheless sufficiently unambiguous representation of real-world concepts, supported of course by common sense knowledge. This is one of the reasons for representing facts and knowledge about a system and its domain-specific subsystems, their architecture and behavior by deploying natural language based domain-specific terminologies and concepts, i.e., domain-specific ontologies, extensively exploited in good modeling best practices. For more information on KR and KM see, e.g., (9, 25, 26).

For modeling pHealth ecosystems, the architectural model (Figure 2A) has to be instantiated by representing its components and relationships using the domain ontologies of the subsystems describing the specific perspectives of the domains involved in the ecosystem including business case specific as well as context specific constraints. For guaranteeing workable knowledge-based interoperability, upper level or foundational ontologies such as the Open Basic Ontology (OBO) and its smaller pendant the Basic Formal Ontology (BFO) as well as agreed international domain ontologies such as the biomedical ontologies Gene Ontology, Cancer Ontology, or SNOMED-CT (28), Policy Ontology (27), domestic environment ontology DomoML-env (29), etc., are deployed for use-case-specifically instantiating the generic pHealth ecosystem. Only within the multi-domain interoperability business view, but not within the different views of ISO 10746 (30) represented using information and communication technology (ICT) ontologies as discussed in more details in the next section, the correctness and consistency of concepts, relations and constraints in pHealth ecosystems can be decided.

## The ISO Interoperability Reference Architecture

The ISO Interoperability Reference Architecture Model (Figure 2B) (31–33) is a generic architecture model, systems-theoretically conceptualized as White-Box, which can be recursively specialized or generalized, respectively. That way, the complexity of the model can be reduced, nevertheless covering the continuum from elementary particles to the universe. The different perspectives of the different disciplines establishing pHealth are represented by domain-specific subsystems. The model representation is mathematically based on the Universal Type Theory including universal logics. Domain-specific views on the considered system are represented by the corresponding domain ontologies. The ISO Interoperability Reference Architecture combines ecosystem models of real-world business systems (Figure 2A) and the development process for complex information and communication technology (ICT) systems according to ISO/IEC 10746 Information technology—Reference model—Open distributed processing (RM-ODP) (30). ISO/IEC 10746 defines and interrelates different perspectives (or views/viewpoints) on the intended system provided by the system designer, the information architect, the component developer, the implementer and finally the system administrator, resulting in the Enterprise View, the Information View, the Computational View, the Engineering View, and the Technology View. It serves as foundations for the Service Oriented Architecture (SOA) (34), the HL7 Development Framework (HDF) (35), or the current HL7 FHIR approach (36) intended to replace HDF. The definition of views or viewpoints on information systems complies with ANSI/IEEE 1471-2000 IEEE Recommended Practice for Architectural Description for Software-Intensive Systems (37). The ISO Interoperability Reference Architecture Framework describes the rules applied to formally represent the concepts and behavioral aspects of, and the relations between, the system's components both inside and between the domains. That way, it enables analysis, design and implementation of multidisciplinary pHealth systems by formally representing and mapping the different involved domains' perspectives. The ISO Interoperability Reference Architecture allows re-engineering of existing specifications and standards.

In 2015, ISO/TC215 and CEN/TC251 decided to mandatorily include the ISO Interoperability Reference Architecture—used in many HL7, ISO, and CEN standards already—in all their revised interoperability specifications. Among others, ISO 13606 Health informatics—EHR communication (33), ISO 12967 Health informatics—Health information service architecture (38), ISO 13940 Health informatics—System of concepts to support continuity of care (39), or ISO/TS 13972 Health informatics—Detailed clinical models, characteristics and processes (40), ISO 22600 Health informatics—Privilege management and access control (41), ISO 21298 Health informatics—Structural and functional roles (42), have to be mentioned here. Additionally, those standards shall contain re-engineered versions of their basic models for the sake of easy and practical interoperability. In the future, all those and newly revised standards will refer to the current project ISO 23903 Health informatics—Interoperability reference architecture (43) model and framework, just providing their re-engineered basic models. Also the aforementioned top level ontologies such as the Basic Formal Ontology are currently turned to ISO standards such as ISO/IEC CD 21838-1 Information technology—Top-level ontologies—Part 1: Requirements (44) and ISO/IEC CD 21838-2 Information technology—Top-level ontologies—Part 2: Basic formal ontology (45).

## Interoperability Challenge of pHealth

**Figure 3:**
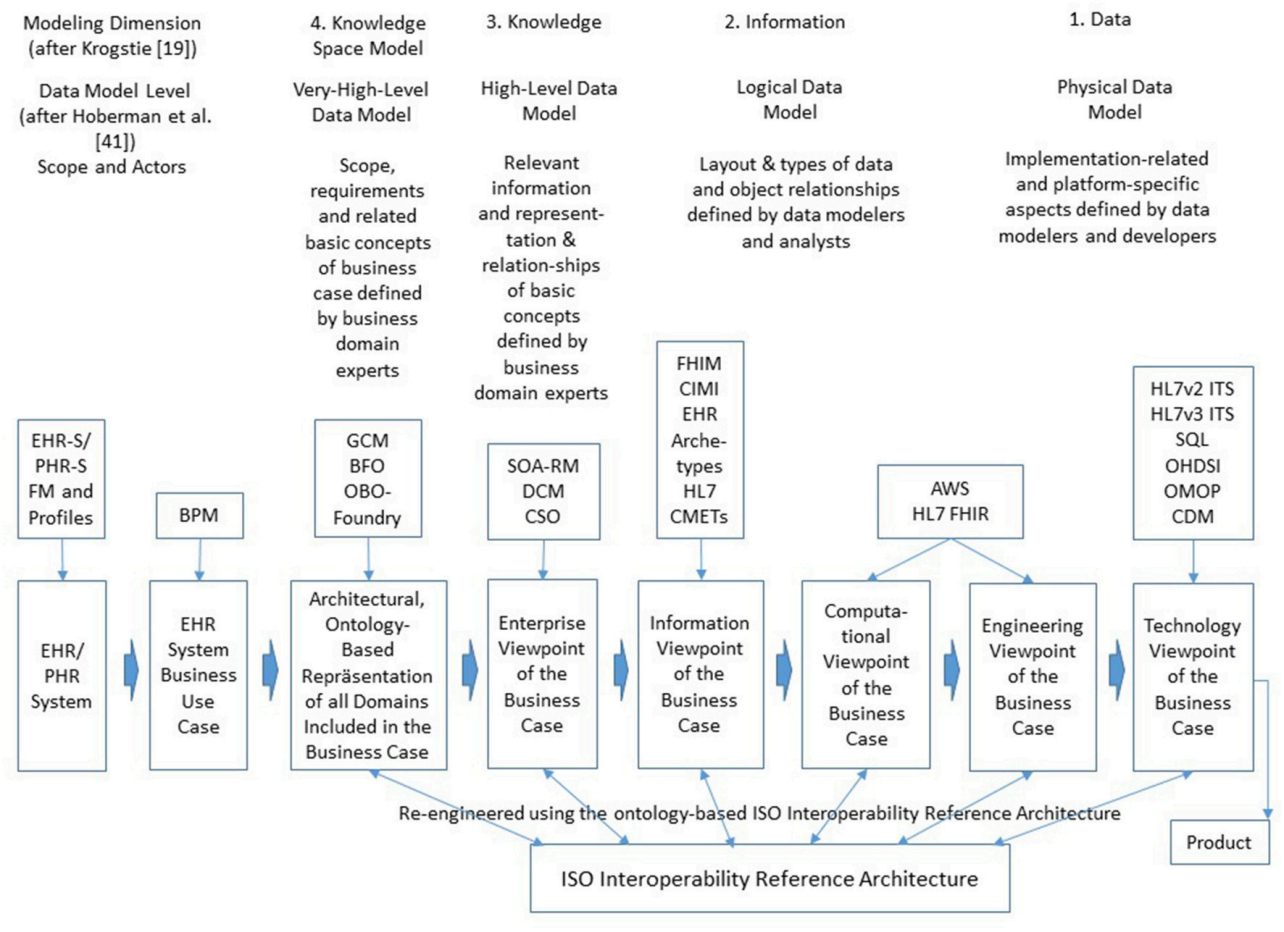
Design and implementation framework for pHealth ecosystems.

There are multiple interoperability definitions in place. The widely used Merriam Webster definition of interoperability as the “ability of a system (as a weapons system) to use the parts or equipment of another system” (46) has been specialized to the IEEE interoperability definition specific for information and communication technologies (ICT) as the “ability of two or more systems or components to exchange information and to use the information that has been exchanged” (47). P5M interoperability has to go far beyond the IT domain, considering motivation, willingness, ability, and capability of all actors involved in the real-world pHealth business system to cooperate for achieving the business objectives, thereby frequently turning the subject of care to the health manager. This requires the harmonization of the actors' knowledge, abilities and skills by sharing and adapting them a-priori or dynamically at runtime to establish adequately cooperating associated systems. As business cases and related policies in pHealth ecosystems cannot be pre-defined, but are determined by the subject of care‘s status, needs, wishes, expectations and its specific context, they require highly dynamic interoperability services provided at real-time. Therefore, pHealth ecosystems' design and implementation have to cover all levels of ICT-related interoperability health informatics standards usually address, i.e., from technical interoperability through structural interoperability, syntactic interoperability, semantic interoperability, and organization/service interoperability. Furthermore, also interoperability beyond ICT-related business cases represented through domain-specific ontologies such as knowledge-based domain-domain interoperability and even skills based interoperability addressing the end-user has to be provided (24, 31). Those different interoperability levels are directly related to different viewpoints of the ISO 10764 Open Distributed Systems Reference Modell (RM-ODP) (30) from Technology through Engineering View, Computational View, and Information View up to Enterprise View, however necessarily extended by the Business View of the pHealth ecosystem. The formally represented real-world multi-domain interoperability business view can then be automatically transformed into the aforementioned views of ISO 10746. Figure 3 demonstrates the system development process for distributed, open systems according to ISO 10746, thereby classifying the models according to their maturity level regarding pHealth ecosystems design and implementation (19, 48). Examples for existing and emerging standards, specifications, and projects related to those viewpoints and their re-engineering using the ISO Interoperability Reference Architecture are especially mentioned. Abbreviations not introduced before are: EHR-S (Electronic Health Record System), PHR-S (Personal Health Record System), FM (Functional Model), BPM (Business Process Modeling), DCM (Detailed Clinical Models), CSO (Communication Standards Ontology), FHIM (Federal Health Information Model), CIMI (Clinical Information Modeling Initiative), CMET (Common Model Element Type), AWS (Amazon Web Service), FHIR (Fast Health Interoperability Resource), ITS (Implementable Technical Specification), SQL (Standard Query Language), OHDSI (Observational Health Data Sciences and Informatics), OMOP (Observational Medical Outcomes Partnership), and CDM (Common Data Model). For more information regarding the standards, specifications, and projects referenced in Figure 3, please refer to Blobel and Oemig (24), Blobel et al. (49).

## Summary

In summary, pHealth ecosystems have to meet the following requirements and characteristics supported by appropriate methodologies and technologies to realize P5M (Table 1).

**Table 1:** PHealth objectives, characteristics and methodologies/technologies to meet objectives (10).

**Objective**	**Characteristics**	**Methodologies/Technologies**
Provision of health services everywhere anytime	• Openness • Distribution • Mobility • Pervasiveness • Ubiquity	• Wearable and implantable sensors and actuators • Pervasive sensor, actuator and network connectivity • Embedded intelligence • Context awareness
Individualization of the system according to status, context, needs, expectations, wishes, environments, etc., of the subject of care	• Flexibility • Scalability • Cognition • Affect and Behavior • Autonomy • Adaptability • Self-organization • Subject of care involvement • Subject of care centration	• Personal and environmental data integration and analytics • Service integration • Context awareness • Knowledge integration • Process and decision intelligence • Presentation layer for all actors
Integration of different actors from different disciplines/do-mains (incl. the participation/empowerment of the subject of care), using their own languages, methodologies, terminologies, ontologies, thereby meeting any behavioral aspects, rules and regulations	• Architectural framework • End-user interoperability • Management and harmonization of multiple domains including policy domains	• Terminology and ontology management and harmonization • Knowledge harmonization • Language transformation/ translation
Usability and acceptability of pHealth solutions	• Preparedness of the individual subject of care Security, privacy and trust framework • Consumerization • Subject of care empowerment • Subject of care as manager • Information based assessment and selection of services, service quality and safety as well as trustworthiness • Lifestyle improvement and Ambient Assisted Living (AAL) services	• Tool-based ontology management • Individual terminologies • Individual ontologies • Tool-based enhancement of individual knowledge and skills • Human Centered Design of solutions • User Experience Evaluation • Trust calculation services

PHealth systems require a system-oriented, architecture-centric, ontology-based, and policy-driven approach. For correctly and consistently designing and implementing pHealth systems, health professionals and other involved domain experts have to play a dominant role. The ISO 23903 Interoperability Reference Architecture approved at ISO/TC 215 and CEN/TC 251 as reference model for any health systems interoperability solution provides the necessary framework. The feasibility of the proposed solution has been demonstrated for different business systems and use cases. Examples are an adaptive, interoperable and intelligent Type 2 Diabetes Mellitus care system (50–52), or the development of a communication standards ontology for automatically interrelating HL7 v2 and HL7 v3 specifications (53). For managing the entire process of defining, designing, specifying, implementing, and deploying the systems regarding ontology representation, management and harmonization, business process modeling, and ICT system design, specification and implementation, Open Source tools have been used. Because of its complex, dynamic nature and personal impacts, appropriate security and privacy solutions are inevitable (27, 54, 55). Here, technologies such as blockchains may come into play. However, this might be an issue of another paper.

## Author Contributions

The author confirms being the sole contributor of this work and has approved it for publication.

### Conflict of Interest Statement

The author declares that the research was conducted in the absence of any commercial or financial relationships that could be construed as a potential conflict of interest.
